# Visual Detection of Chicken Adulteration Based on a Lateral Flow Strip-PCR Strategy

**DOI:** 10.3390/foods11152351

**Published:** 2022-08-05

**Authors:** Haoyi Xu, Hangzhen Lan, Daodong Pan, Junfeng Xu, Xiaofu Wang

**Affiliations:** 1 State Key Laboratory for Managing Biotic and Chemical Threats to the Quality and Safety of Agro-Products, Ningbo University, Ningbo 315211, China; 2 Key Laboratory of Animal Protein Deep Processing Technology of Zhejiang Province and College of Food and Pharmaceutical Sciences, Ningbo University, Ningbo 315800, China; 3 State Key Laboratory for Managing Biotic and Chemical Threats to the Quality and Safety of Agro-Products, Zhejiang Academy of Agricultural Sciences, Hangzhou 310021, China; 4 Key Laboratory of Traceability for Agricultural Genetically Modified Organisms, Ministry of Agriculture and Rural Affairs, Hangzhou 310021, China

**Keywords:** chicken, meat adulteration, DNA extraction, polymerase chain reaction, lateral flow strip

## Abstract

The aim of this study was to develop an accurate, easy-to-use, and cost-effective method for the detection of chicken adulteration based on polymerase chain reaction (PCR) and lateral flow strip (LFS). We compared six DNA extraction methods, namely the cetyltrimethylammonium bromide (CTAB) method, salt method, urea method, SDS method, guanidine isothiocyanate method, and commercial kit method. The chicken cytb gene was used as a target to design specific primers. The specificity and sensitivity of the PCR-LFS system were tested using a self-assembled lateral flow measurement sensor. The results showed that the DNA concentration obtained by salt methods is up to 533 ± 84 ng µL^−1^, is a suitable replacement for commercial kits. The PCR-LFS method exhibits high specificity at an annealing temperature of 62 °C and does not cross-react with other animal sources. This strategy is also highly sensitive, being able to detect 0.1% of chicken in artificial adulterated meat. The results of the test strips can be observed with the naked eye within 5 min, and this result is consistent with the electrophoresis result, demonstrating its high accuracy. Moreover, the detection system has already been successfully used to detect chicken in commercial samples. Hence, this PCR-LFS strategy provides a potential tool to verify the authenticity of chicken.

## 1. Introduction

Meat products are an important source of nutrition for the human body. As people’s living conditions and quality of life increase, the demand for meat products is increasing. However, food fraud is rampant, and food-safety issues have become increasingly concerning. Just like the horsemeat fiasco in Europe in 2013, unscrupulous merchants sold horsemeat disguised as beef [1]. In addition, processed meat products (such as ham sausage, bacon, meatballs, etc.) made from ground meat have the most serious problem of counterfeiting [2]. The merchants gain excessive profit by adulterating low-cost chicken, duck, and pork into high-priced beef and lamb [3,4]. The above phenomenon not only deprives consumers of the right to know and access quality goods but also disrupts the economic order of the market. As a consequence, it is critical to develop simple, accurate, and efficient technology to identify the adulteration of meat products.

At present, methods of meat composition identification are mainly based on protein and nucleic acid analysis [5]. Protein-based methods for meat component identification include enzyme-linked immunosorbent assays (ELISA) [6], spectroscopy [7,8], mass spectrometry [2,9], chromatography [10], and western blot [11]. However, proteins are easily denatured during meat pre-treatment and processing, leading to poor reproducibility and low detection sensitivity [12]. Nucleic acids show greater thermal and physical stability than proteins, which makes them more suitable for the identification of adulterated meat products. Furthermore, the amplification capacity of nucleic acids can improve the detection sensitivity of corresponding DNA analytical methods. Commonly used nucleic acid-based detection methods include polymerase chain reaction (PCR) [13], real-time fluorescent PCR [14], digital PCR [15], multiplex PCR [16], loop-mediated isothermal amplification (LAMP) [17], cross-priming amplification (CPA) [18], and recombinase polymerase amplification (RPA) [19,20]. PCR is considered the “gold standard” for identifying adulterated meat products and has been successfully employed to identify beef, lamb, pork, and chicken [21]. However, PCR results need to be analyzed by electrophoresis, and it is difficult to quantify the amplicons. Although real-time quantitative fluorescence PCR and digital PCR have addressed this issue, they require expensive instruments and specialized operations, making them inapplicable to developing countries and resource-limited areas.

The colloidal gold lateral flow sensor is a new immunolabelling technique. The test is achieved by the specific binding of antigens and antibodies with colloidal gold as the tracer [22]. It is simple, fast, non-polluting, user-friendly, and has been widely used for the identification of meat adulteration [23,24]. Chen et al. combined PCR technology with flow test strips to establish a Horse-PCR-LFS sensor for rapid detection of horse meat [25]. Qin et al. combined multiplex polymerase chain reaction with lateral flow strip to simultaneously detect chicken, duck, and pork adulteration in beef [4]. The combination of polymerase chain reaction and test strip technology provides an important tool for the identification of meat products. In this research, we developed a PCR-LFS strategy to identify chicken components in high-priced meats, e.g., beef, lamb, and pork. First, several literatures reported and commercial DNA extraction methods were compared in order to quickly obtain high-purity DNA at low cost. Then, the specific forward primer and reverse primer of the chicken cytb gene were designed and modified with Fam and biotin, respectively, on the 5’ end to achieve a binding reaction between the amplificants and gold nanoparticles (located on the conjugate pad) and antibodies (located on the test line). To the end, a lab-made LFS was fabricated and employed for the semi-quantitative detection of chicken DNA amplificants with the naked eyes. This strategy provides a simple, cost-effective, visual, and highly specific method for detecting chicken adulteration in animal-origin food.

## 2. Materials and Methods

### 2.1. Materials and Reagents

Ethylene diamine tetraacetic acid (EDTA), cetyltrimethylammonium bromide (CTAB), sodium dodecyl sulfate (SDS), Tris-HCl, NaCl, urea, Na_2_CO_3_, NaHCO_3_, sodium citrate (C_6_H_5_Na_3_O_7_), guanidine isothiocyanate (C_2_H_6_N_4_S), sodium N-dodecanoylsalcosinate (C_15_H_10_NO_4_Na), β-mercaptoethanol, alcohol, isoamyl alcohol, and isopropanol were purchased from Sangon Biotech (Shanghai, China). HAuCl_4_ was purchased from Sinopharm (Shanghai, China). Bovine serum albumin (BSA), sucrose, and alginate were obtained from Meilunbio Biotech (Dalian, China). Absorbent pads (CH27) and sample pads (SB06) were purchased from KinBio Biotech (Shanghai, China). The gold absorbent pad (GL0194), PVC backing plate (DB-6), and nitrocellulose membrane (Millipore 135) were obtained from Jiyi Biotech (Shanghai, China). PCR Master Max and the anti-5-FAM antibody were obtained from BBI Biotech (Shanghai, China). Streptavidin and biotin-BSA were obtained from Solarbio Biotech (Beijing, China).

### 2.2. Meat Samples

Seven fresh meat samples (chicken, duck, pork, beef, lamb, horse, and ostrich) and 48 processed meat samples (beef rolls, lamb rolls, beef skewers, lamb skewers, and meatballs) were acquired from local markets in Ningbo, China. The purchased meat samples were rinsed with distilled water, ground in a meat grinder, and stored in sealed bags at −20 °C. To simulate chicken adulteration, we mixed different proportions of chicken meat with beef, lamb, and ostrich meat (Table 1), and were used for subsequent sensitivity detection experiments.

### 2.3. DNA Extraction

**Figure 1:**
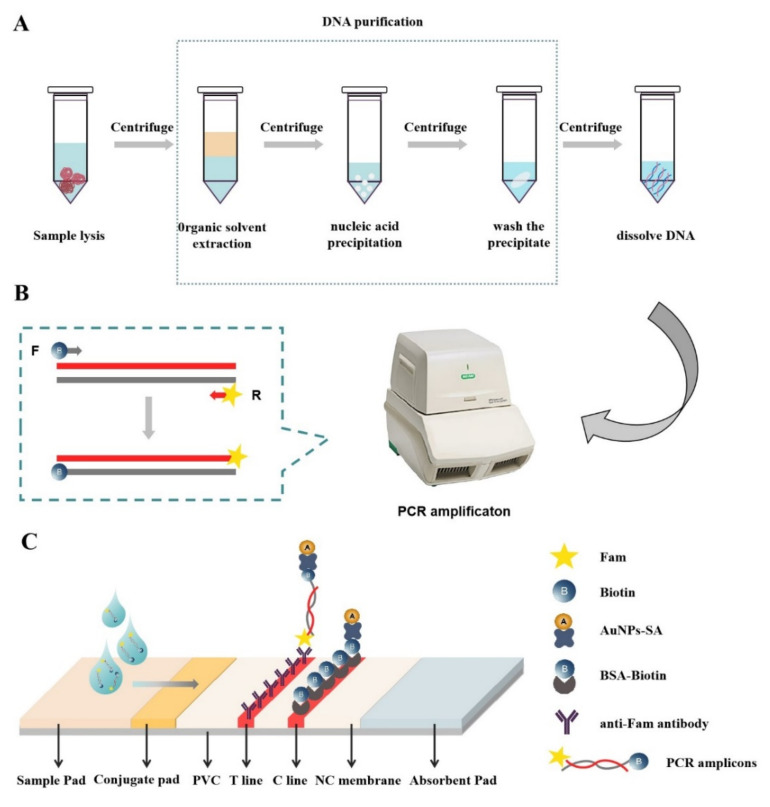
Schematic diagram of chicken adulteration detection based on the PCR-LFS strategy. (**A**) DNA extraction and purification; (**B**) PCR amplification; (**C**) Test strip detection.

Six different methods were used to extract DNA from the chicken samples, and three replicate experiments were carried out for each method. DNA is extracted in five steps: sample lysis, organic solvent extraction, nucleic acid precipitation, wash the precipitate, and dissolve DNA (Figure 1A). The DNA concentration was measured using NanoDrop 2000. The highest absorption peaks at 260 nm, 280 nm, and 230 nm were measured by spectrophotometer, and the ratio of A_260_/A_280_ and A_260_/A_230_ to determine the purity of the DNA.

#### 2.3.1. CTAB Method

The CTAB method was performed according to the literature, with slight modifications [26]. Thirty milligrams of the meat sample was mixed with 800 µL lysate buffer (25 mM CTAB, 60 mM Tris-HCl, 15 mM EDTA, 100 mM NaCl, pH = 8.0) and incubated at 65 °C for 30 min after adding 2 µL of proteinase K (20 mg mL^−1^). Then, the mixture was centrifuged for 5 min at 13,000× *g*, and the supernatant was transferred to a new tube with 400 µL of trichloromethane-isoamyl alcohol (24:1, m/m). Thereafter, the solution was centrifuged for another 5 min at 13,000× *g*, and the supernatant was mixed with 400 µL of isopropanol. The mixture was kept at room temperature for 45 min and then centrifuged at 13,000× *g* for 5 min. The precipitant was washed with ethanol/water solution (70%, *v*/*v*). The ethanol was discarded after centrifugation, and the residual ethanol was evaporated at ambient temperature. Finally, the precipitated DNA was dissolved in TE buffer.

#### 2.3.2. Salt Method

The salt method was performed according to the literature, with slight modifications [27]. Thirty milligrams of the meat sample was mixed with 500 µL lysis buffer (400 mM NaCl, 10 mM Tris-HCl, 20 mM EDTA, Ph = 8.0) and incubated at 65 °C for 45 min after adding 50 µL of 20% SDS and 2 µL of Proteinase K (20 mg mL^−1^). Then 400 µL of 6 M NaCl was added and mixed upside down. The mixture was centrifuged at 13,000× *g* for 5 min, and the supernatant was mixed with 500 µL of isopropanol. Thereafter, the solution was centrifuged for another 5 min at 13,000× *g*, and the precipitate was washed with ethanol/water solution (70%, *v*/*v*). The ethanol was discarded after centrifugation, and the residual ethanol was evaporated at an ambient temperature. Finally, the precipitated DNA was dissolved in TE buffer.

#### 2.3.3. Urea Method

The urea method was carried out according to the literature, with slight modifications [27]. Thirty milligrams of the meat sample was mixed with 500 µL lysis buffer (8 M urea, 1% SDS, 10 mM Tris-HCl, 20 mM EDTA, pH = 8.0) and incubated at 60 °C for 20 min after adding 2 µL of Proteinase K (20 mg mL^−1^). Then, 50 µL of 5 M NaCl and 500 µL of phenol/chloroform/isoamyl alcohol (25:24:1, m/m/m) were added to the purified DNA. The mixture was centrifuged at 13,000× *g* for 5 min, and the supernatant was removed in 500 µL of isopropanol. Thereafter, the solution was centrifuged for another 5 min at 13,000× *g*, and the precipitate was washed with ethanol/water solution (70%, *v*/*v*). The ethanol was discarded after centrifugation, and the residual ethanol was evaporated at an ambient temperature. Finally, the precipitated DNA was dissolved in TE buffer.

#### 2.3.4. SDS Method

The SDS method was performed according to the literature, with slight modifications [28]. Thirty milligrams of the meat sample was mixed with 500 µL lysis buffer (2% SDS, 100 mM NaCl, 10 mM Tris-HCl, 25 mM EDTA, Ph = 8.0) and incubated at 60 °C for 20 min after adding 2 µL of Proteinase K (20 mg mL^−1^). An equivalent amount of Tris-saturated phenol was added and mixed upside down. Then, the mixture was centrifuged for 5 min at 13000× *g*. Extraction was carried out with an equal volume of chloroform/isoamyl alcohol (24:1, m/m). Thereafter, the solution was centrifuged for another 5 min at 13,000× *g*, and the supernatant was mixed with 500 µL of isopropanol. The mixture was kept at −20 °C for 2 h and then centrifuged at 13,000× *g* for 5 min. The precipitate was washed with ethanol/water solution (70%, *v*/*v*). Subsequently, ethanol was discarded after centrifugation, and the residual ethanol was evaporated at an ambient temperature. Finally, the precipitated DNA was dissolved in TE buffer.

#### 2.3.5. Guanidine Isothiocyanate Method

The guanidine isothiocyanate method was performed according to the literature with slight modifications [28]. Thirty milligrams of the meat sample was mixed with 500 µL lysis buffer (2.5 M C_2_H_6_N_4_S, 25 mM C_6_H_5_Na_3_O_7_, 20 mM C_15_H_10_NO_4_Na, 100 mM β-mercaptoethanol, 20 mM CBS buffer). and incubated at 60 °C for 20 min after adding 2 µL of Proteinase K (20 mg mL^−1^). Then, 300 µL of chloroform/isoamyl alcohol (24:1) and 300 µL of Tris-saturated phenol were added to the purified DNA. Then, the mixture was centrifuged for 5 min at 13,000× *g*, and the supernatant was mixed with 500 µL of chloroform/isoamyl alcohol (24:1, m/m). Thereafter, the solution was centrifuged for another 5 min 13,000× *g*, and the supernatant was mixed with 500 µL of isopropanol. Centrifuge at 13,000× *g* for 5 min, and the precipitate was washed by ethanol/water solution (70%, *v/v*). Ethanol was discarded after centrifugation, and the residual ethanol was evaporated at an ambient temperature. Finally, the precipitated DNA was dissolved in TE buffer.

#### 2.3.6. Tiangen Method

The specific operation process of the Tiangen method was performed according to the TIANamp Genomic DNA Kit manual (Cat. No. DP180123, Beijing, China). Take 30 mg of animal tissue in 200 µL of GA buffer and shake it to suspension. Add 20 µL proteinase K and place at 56 °C for 2 h until the tissue is lysed. After adding 200 µL of buffer GB, mix well and leave at 70 °C for 10 min. Then, 200 µL of anhydrous ethanol was added and mixed well. Transfer the solution from the previous step into the adsorption column CB3 (column in a collection tube), centrifuge at 12,000 rpm for 30 s, and discard the waste liquid. Add 600 µL of rinse solution PW to the adsorption column CB3, centrifuge at 12,000 rpm for 30 s, and discard the waste liquid. Repeat the previous step again. Centrifuge for another 2 min at 12,000 rpm to remove the residual rinse solution, and leave the sample at room temperature for a few minutes to thoroughly dry the residual rinse solution on the adsorbent material. Finally, transfer the adsorption column to a clean centrifuge tube, drop 100 µL of TE buffer on the adsorption membrane, placed at room temperature for 5 min, centrifuged, and collected the DNA solution.

### 2.4. Primer Design

A partial sequence of the chicken cytb gene was downloaded from GenBank (Accession No.: L07520.1). Primer Premier 6 was then used to design specific primers. During the design process, extreme caution was exercised to avoid the creation of complicated secondary structures between primers, which would probably result in false-positive test results. The nucleic acid alignment program “BLAST” (Available online: https://www.ncbi.nlm.nih.gov/tools/primer-blast (accessed on 25 July 2022)) was further used to assess primer specificity. The forward primer (FR) was 5′-Biotin-CATGACCCAAATCCTCACCG-3′ (20 bp, Tm: 62 °C), where the 5′ end was modified with biotin; the reverse primer (PR) was 5′-Fam-TCCTTGTAGAGGTAGGAGCCGTA-3′ (23 bp, Tm: 62 °C), where the 5′ end was grafted with Fam. The above primers were all produced by Hangjing Biotech Co., Ltd. (Ningbo, China).

### 2.5. PCR Amplification

The PCR amplification system consisted of 1 µL of forward primer (10 µmol L^−1^), 1 µL of reverse primer (10 µmol L^−1^), 1 µL of template DNA, 10 µL of PCR Master Mix, and 12 µL of ddH_2_O. The PCR amplification program was as follows: pre-denaturation was first carried out at 95 °C for 5 min, followed by denaturation at 95 °C for 30 s, annealing was performed at 62 °C for 30 s and, extension at 72 °C for 30 s (35 cycles). ddH_2_O was used instead of DNA as a negative control. Amplificants were kept at 4 °C before gel electrophoresis analysis (140 V for 30 min).

### 2.6. Synthesis of AuNPs and AuNPs-SA

Colloidal gold particles (AuNPs) were synthesized using the conventional trisodium citrate reduction method [29]. One milliliter of 1% chloroauric acid was mixed with 99 mL of deionized water and stirred at 500 r/min until boiling. Subsequently, 2 mL of 1% trisodium citrate was added and boiled for 15 min. When the color of the solution changed from golden yellow to orange-red, the heating was stopped, and the solution was cooled to ambient temperature. Then, the solution was refilled with deionized water to its original volume, and the synthesized AuNPs were stored at 4 °C. Before labeling with streptavidin (SA), the pH of the AuNPs should be adjusted to 7.0 to approach the isoelectric point of the protein. Then, different amounts (5 µg, 10 µg, 15 µg, 20 µg, 25 µg) of streptavidin (1 mg mL^−1^) were added per ml of colloidal gold solution to determine the optimal amount for protein labeling on AuNPs. The AuNPs-SA composites were stored at 4 °C before further use. The colloidal gold particles were characterized by UV spectrophotometry and transmission electron microscopy (TEM).

### 2.7. Test Strip Fabrication

The test strip was composed of five parts: the sample pad, gold absorption pad, nitrocellulose membrane, absorbent pad, and PVC backing plate. The gold absorbent pads were sprayed with the pretreatment solution (5% BSA, 10% alginate, 2% sucrose, 2% Tween-20, and 2% TritionX-100) and dried at 40 °C for 2 h [30]. The pretreated gold standard pads were then immersed in a colloidal gold protein solution and dried at 40 °C for 2 h. At the same time, 1 mg mL^−1^ of anti-5-Fam antibody and 1 mg mL^−1^ of BSA-biotin were sprayed separately on the nitrocellulose membrane as the test line and the control line, respectively. Finally, all the above-mentioned pads were sticked together and cut into 4 mm in width. The obtained test strips were placed in a sealed container with a desiccant and stored at room temperature before further use.

### 2.8. PCR-LFS Assay

The mechanism for detecting chicken adulteration using the PCR-LFS strategy is shown in Figure 1. The conserved sequence of the chicken cytb gene was used to design specific primers, ensuring that the primer dimers did not cause false positives. The 5′ ends of the forward and reverse primers were labeled with Fam and Biotin, respectively (Figure 1B). After 35 cycles of PCR amplification, the amplicons were placed on the sample pad of the flow measurement sensor. If the sample contained chicken components, the amplification product would contain large amounts of labeled DNA (Fam-DNA-Biotin). The labeled DNA (Fam-DNA-Biotin) first binds to AuNPs-SA on the gold absorption pad and is captured by anti-5-Fam antibodies on the nitrocellulose membrane to form a sandwich structure (anti-5-Fam antibody/Fam-DNA-Biotin/AuNPs-SA), making the T-line red. The remaining AuNPs-SA bound to BSA-Biotin on the nitrocellulose membrane, causing the C line to show red (Figure 1C). In the absence of chicken components, no color appears on the T line of the test strip, while the C line is red. The test strip detection can be completed in less than 5 min, and can be semi-quantified by the naked eye.

### 2.9. Specificity and Sensitivity Detection

The genomic DNA of chicken, duck, pig, beef, lamb, horse, and ostrich was extracted using the salt method and used to determine the specificity of the PCR-LFS system. The sample DNA was amplified using four different annealing temperatures (58, 60, 62, and 65 °C), with ddH_2_O as the negative control.

Three different types of meat adulteration were simulated to test the sensitivity of the PCR-LFS method. To replicate real-life chicken adulteration, different percentages of chicken (0.1, 1, 10, and 100%) were mixed with beef, lamb, and a mixture of beef, lamb, and ostrich.

### 2.10. Statistical Analysis

DNA concentration and purity were determined using NanoDrop 2000, and the mean and standard deviation were calculated using Microsoft Excel software (Microsoft Inc., Washington, DC, USA). Origin software (Origin Lab, Northampton, MA, USA) was used to plot histograms to analyze the relationship between different DNA extraction methods (x) and concentration (y). Significant differences in the DNA concentrations extracted by the different methods were analyzed by a one-way analysis of variance (ANOVA) using GraphPad Prism 8 (GraphPad Software, San Diego, CA, USA), with *p* < 0.05 being a significant difference. The Malvern particle size analyzer was used to measure the diameter of nanoparticles, histograms of the size distribution were plotted using Origin, and the histograms were fitted using a Gaussian equation.

## 3. Results and Discussion

### 3.1. DNA Concentration and Purity 

**Figure 2:**
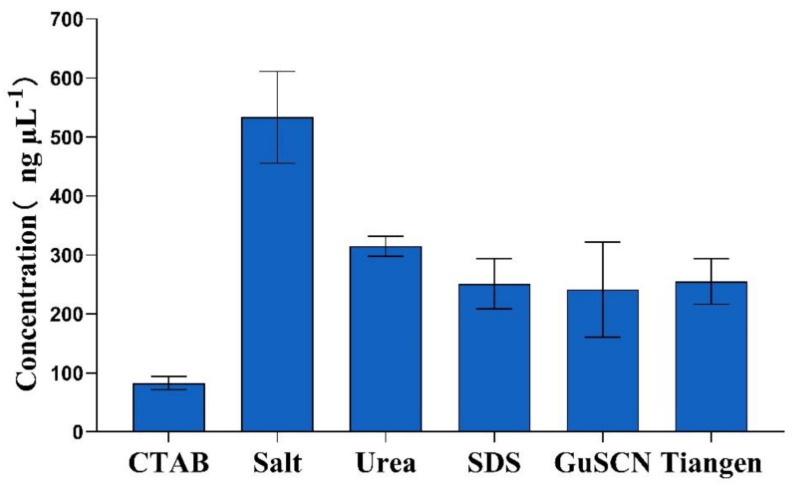
The concentration of DNA extracted by different methods.

A variety of DNA extraction methods have been developed, but each has different effects on meat products, and it is important to compare these methods. The concentration of DNA was measured using NanoDrop 2000. The results showed that the DNA concentrations extracted by the CTAB, salt, urea, SDS, guanidine isothiocyanate, and kit methods were 70 ± 9.7 ng µL^−1^, 533 ± 84 ng µL^−^^1^, 314 ± 13 ng µL^−1^, 251 ± 58 ng µL^−1^, 241 ± 75 ng µL^−1^, and 289 ± 11 ng µL^−1^, respectively (Table 2). The salt method yielded the highest concentration, followed by the urea method and the commercial kit method. In contrast, the CTAB method extracted the lowest concentration of DNA (Figure 2). After multiple comparisons, we found a significant difference between the salt method and the CTAB, SDS, and guanidine isothiocyanate methods, indicating the high concentration of DNA obtained by the salt method (Table 2). One study compared several different fish DNA extraction methods, with the salt method being the most effective [27], which is similar to the results of this study. The concentration of chicken DNA extracted by the salt method in this study was as high as 533 ± 84 ng µL^−1^.

The purity of the DNA was determined by measuring the UV absorbance at 230 nm, 260 nm, and 280 nm. When the ratio of A_260_/A_230_ is between 2.0 and 2.2, and the ratio of A_260_/A_280_ is between 1.8 and 2.0, it can be determined that the DNA is pure. As shown in Table 2, the ratio of A_260_/A_280_ for the six DNA extraction methods ranged from 1.5 ± 0.03 to 2.0 ± 0.09. The ratio for the CTAB method was 1.5 ± 0.03, which was below 1.8, indicating that the extract was contaminated. The ratio of A_260_/A_230_ ranged from 0.5 ± 0.1 to 2.8 ± 0.82, while the ratio for the CTAB method was 0.5 ± 0.1, which was below 2.0. A_260_, A_280_ and A_230_ represent the highest absorption wavelengths for nucleic acids, proteins, and carbohydrates, respectively. Residual proteins or phenols in the sample will cause the A_260_/A_280_ ratio to decrease, and residual carbohydrates and salts in the sample will cause the A_260_/A_230_ ratio to decrease [27,31]. The results suggested that the DNA extracted by several methods had high purity, except for the CTAB method. Nucleic acid is a powerful molecular tool, but the extraction process is extremely tedious, limiting its use in out-of-lab settings. A variety of rapid DNA extraction methods have been developed and used. There is a study using nucleic acid extraction test strips to purify nucleic acids from animals, plants, and microorganisms within 30 s [32]. Although the purity of the DNA extracted using the above method was poor, it could be successfully amplified by PCR. Therefore, in subsequent experiments, we can combine rapid DNA extraction techniques with PCR to achieve rapid detection. In summary, the salt method offers the highest extraction concentration, good purity, simple operation, cost-effectiveness, and environmental friendliness, making it a suitable alternative method for extracting meat DNA.

### 3.2. Characterization of AuNPs

**Figure 3:**
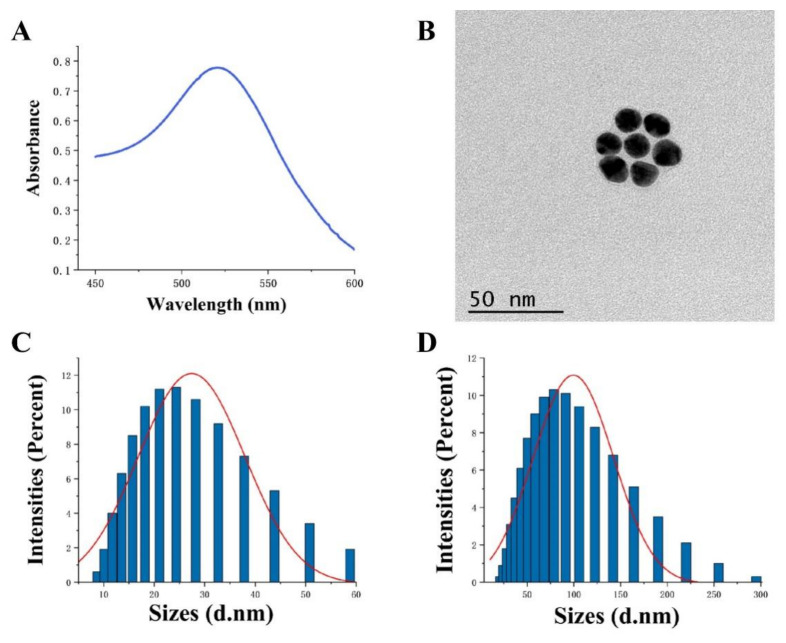
(**A**) UV absorption spectra of AuNPs; (**B**) TEM images of AuNPs; (**C**,**D**) represent the particle size distribution of AuNPs and AuNPs-SA, respectively.

Gold nanoparticles have been selected as signal molecules for flow measurement sensors due to their high stability, ease of synthesis, strong colorimetric signal, and high protein adsorption capacity [33]. The colloidal gold solution was prepared using the trisodium citrate reduction method [29]. The solution was transparent and free of agglutinated particles under natural light. The UV spectrogram showed that the colloidal gold had the highest absorption peak (λ_max_ = 524 nm) between 450 nm and 600 nm (Figure 3A). According to the linear regression equation Y (λ_max_) = 0.4271 × (particle diameter) + 514.56, the preliminary calculation of the colloidal gold particle diameter was 18 nm, and the results of this particle size were consistent with previous studies [34]. Transmission electron microscopy revealed that the colloidal gold particles were uniformly dispersed, spherical, with no polygons and an average diameter of about 18 nm (Figure 3B), which was consistent with the UV detection results. The results showed that the colloidal gold solution was stable and reliable and could meet the experimental needs well. The particle size distribution of colloidal gold and colloidal gold protein was measured using a nanoparticle analyzer. The colloidal gold particles were normally distributed between 10 nm and 60 nm, with an average diameter of 18 nm (Figure 3C). Therefore, the colloidal gold particles were of uniform size and high quality and were suitable for subsequent protein labeling. The particle size of the colloidal gold protein was normally distributed between 20–250 nm, with an average diameter of 65 nm (Figure 3D), indicating that the colloidal gold was successfully labeled with streptavidin.

### 3.3. Optimization of Lateral Flow Strips

**Figure 4:**
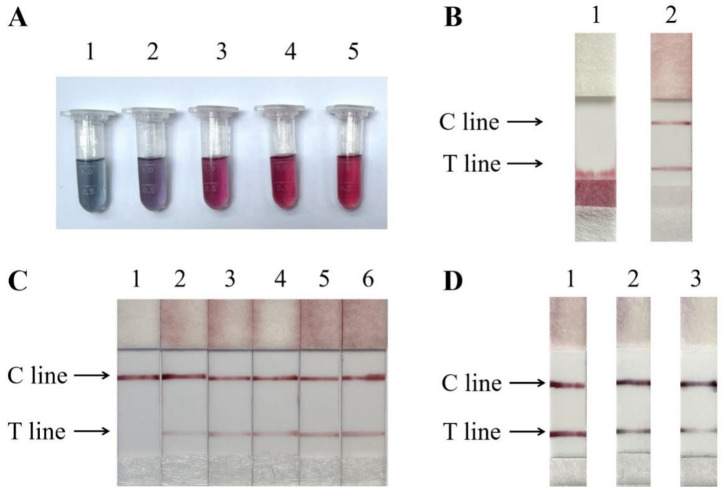
(**A**) Optimal streptavidin labeling of AuNPs, from left to right, is 5 µL, 10 µL, 15 µL, 20 µL and 25 µL. (**B**) Optimization of gold absorbent pads. (**C**) Optimization of loading volume, from left to right, is negative control (ddH_2_O), 2.5 µL, 5 µL, 10 µL, 15 µL and 20 µL. (**D**) Optimization of AuNPs diameter. From left to right, 20 nm, 30 nm, and 40 nm.

Before obtaining AuNPs-SA conjugates, we need to select the optimal amount of streptavidin to label the AuNPs. Accurate amounts of protein labeling ensure the stability of immune complexes. The results showed that the tubes exhibited aggregation from red to blue when the amount of colloidal gold-labeled protein was 5 µg, 10 µg and 15 µg per ml. The colloidal gold solution remains red when the protein labeling amount is 20 µg and 25 µg (Figure 4A). Insufficient amounts of protein will lead to colloidal gold particle surface instability to cause aggregation, which can be seen as colloidal gold solution from red to blue [35]. Therefore, 20 µg of streptavidin is sufficient to stabilize 1 mL of colloidal gold solution, which can be appropriately increased by 10% as the optimum protein labeling amount in practice. The gold absorbent pads determine the sensitivity, specificity, release effect, and long-term stability of AuNPs-SA, and therefore have a significant impact on the effectiveness of colloidal gold test strips. According to the results, the untreated gold absorbent pads failed to release gold particles and could not bind to the test and control lines to form a sandwich complex. After pretreatment, the colloidal gold was released completely without residual particles, and the test line and quality control line were successfully colorized (Figure 4B). The presence of surfactant and blocking agent in the pretreatment solution not only protects the activity of the colloidal gold protein but also accelerates its release, improving the speed and reliability of the assay. The optimal loading volume was determined by testing different volumes (0 µL, 2.5 µL, 5 µL, 10 µL, 15 µL and 20 µL) of chicken amplification products. The color of the T-line gradually deepens with the increase in the sample volume, and the insufficient amount of the upper sample will result in the T-line not being obvious (Figure 4C). For this reason, we took 15 µL of the product for the test strip assay in subsequent experiments. Finally, three types of colloidal gold particles of 20, 30, and 40 nm are prepared for diameter optimization. The best color development was achieved when the diameter of the test strip was 20 nm. As the diameter increases, the color rendering of the test strips becomes less effective. (Figure 4D). As the diameter of colloidal gold increases, its diffusion rate on the nitrocellulose membrane decreases, and the affinity between the colloidal gold complex and the antibody on the test line decreases, ultimately reducing the sensitivity of the test strips [22].

### 3.4. Specificity of the Test Strip Method

**Figure 5:**
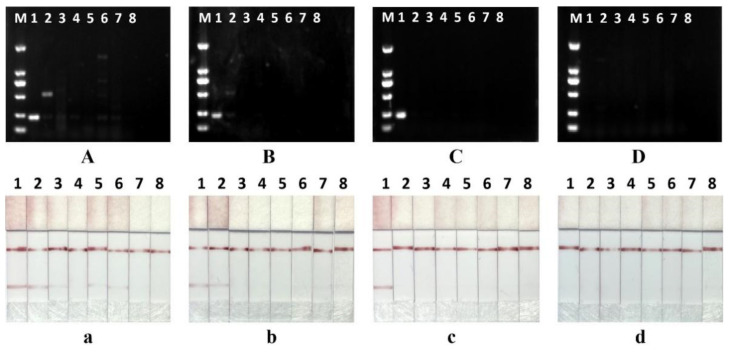
Specificity of the PCR-LFS assay; 1–8 are chicken, duck, pork, beef, lamb, horse, ostrich, and negative control (ddH_2_O), respectively. (**A**)(**a**), (**B**)(**b**), (**C**)(**c**), and (**D**)(**d**) were obtained at annealing temperatures of 58 °C, 60 °C, 62 °C, and 65 °C, respectively.

The specificity of the PCR-LFS system was assessed using seven different meat samples (chicken, duck, pig, beef, lamb, horse, and ostrich). According to the test results, the non-specific products were present in lanes 2 and 6 when the annealing temperature was 58 °C (Figure 5A), which would result in false-positive test strip results (Figure 5a). When the annealing temperature was increased to 60 °C, the non-specific amplicons were reduced (Figure 5B). However, there were still non-specific products in lane 2, which made the test strip detection unspecific (Figure 5b). The detection system showed high specificity at the annealing temperature of 62 °C (Figure 5C) and the results of the test paper were consistent with those of electrophoresis (Figure 5c). There were no specific amplicons at the annealing temperature of 65 °C (Figure 5D). In a certain range, the specificity of PCR amplification increases with an increase in annealing temperature. Too high an annealing temperature can lead to poor primer binding to the DNA template and hinder amplification [36]. In addition, the selection of target genes and the design of specific primers play decisive roles in the identification of meat products. The cytochrome b (cytb) gene has a high rate of evolutionary variation and interspecific variation and is widely used for species identification [37]. PCR amplification based on simultaneous 5′ tagging of forward and reverse primers has limits. Primer dimers can compete with positive amplicons and cause false-positive results. Therefore, extra care should be taken during the design of primers to avoid the formation of secondary primer structures. According to the electrophoresis results in Figure 5, there are no secondary structures of primers, and non-specific amplicons can also be resolved by increasing the annealing temperature. Some studies have introduced specific probes with markers to hybridize with denatured PCR amplicons for test strip detection, to improve species specificity, and to address the problem of false positives caused by primers [12,25]. However, the method adds additional denaturation and probe hybridization steps, increasing the complexity of the experiment. The PCR-LFS strategy based on this study was extremely specific at an annealing temperature of 60 °C, and the amplified products could be used directly for test strip identification.

### 3.5. Sensitivity of the Test Strip Method

**Figure 6:**
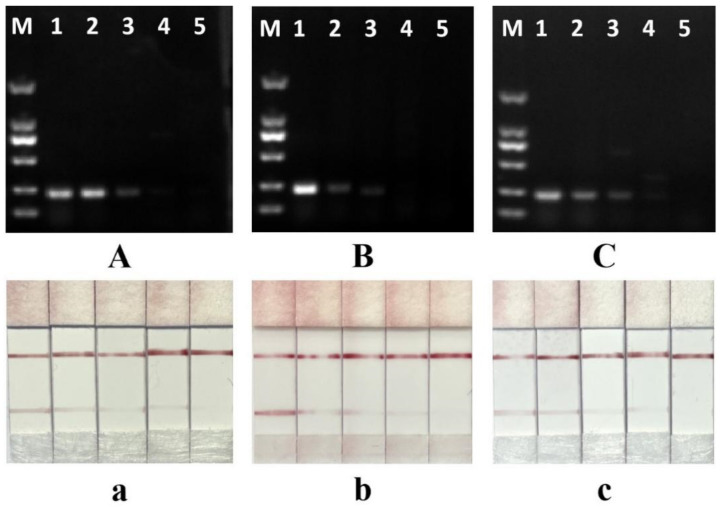
Sensitivity of the PCR-LFS assay. The mixed meat contains different propotions of chicken. Numbers 1–5 represent 100%, 10%, 1%, 0.1% of chicken content and the negative control, respectively. (**A**)(**a**): Simulated beef mixed with chicken; (**B**)(**b**): simulated lamb mixed with chicken; (**C**)(**c**): simulated beef lamb and ostrich mixed with chicken.

Chicken was blended into beef, lamb, and a mixture of beef, lamb, and ostrich at rates of 0.1%, 1%, 10%, and 100% (Table 1) to verify the sensitivity of the PCR-LFS strategy. As shown in Figure 6a,b, the PCR-LFS can detect 0.1% (*w*/*w*) of chicken in beef and lamb, and the color of the test strip becomes lighter as the adulteration ratio decreases. However, gel electrophoresis detected only 1% of the chickens in adulterated meat (Figure 6A,B). Hence, PCR-LFS is more sensitive than PCR-electrophoresis, which is consistent with the previous comparison between Pig-PCR-LFS and Pig-PCR-electrophoresis [12]. As shown in Figure 6c, the system can detect 0.1% chicken in multiple meat mixtures, which is consistent with single-component adulteration. This shows that the system can adapt to complex adulteration environments and has strong practical application ability. A study established a colloidal gold immunochromatographic method for the detection of chicken in food using chicken immunoglobulin (IgY) as a target, but the method has poor thermal stability and has a detection limit of 5% in processed meat products [38]. However, this study is based on the nucleic acid level and has a high stability and a detection limit of 0.1%, which makes it more suitable for detecting chicken adulteration. According to reports, if the level of adulteration in meat products is less than 0.1%, it is not economically viable and can be considered the result of accidental contamination [39]. Therefore, we did not conduct further sensitivity tests with lower adulteration ratios. PCR-based flow measurement sensors achieve signal amplification through DNA amplification. Traditional colloidal gold test strips have low sensitivity and only allow for qualitative testing, not quantitative testing. To improve the sensitivity of the test strips, the signal strength of the probe can be enhanced, or the affinity of the complex to the detection line can be increased [40]. Li et al. loaded many quantum dots (QBs) into large-size polymer beads as signal probes to improve the sensitivity of the immunoassay [40]. Li et al. assembled gold nanoparticles in a three-dimensional manner on silica scaffolds as signal reporters [41]. The above methods not only allow qualitative analysis of low concentration targets but also achieve ultra-sensitive quantitative detection with an optical scanner or smartphone and offer a potential avenue for point-of-care (POC) detection [42,43].

### 3.6. Meat Sample Detection

**Figure 7:**
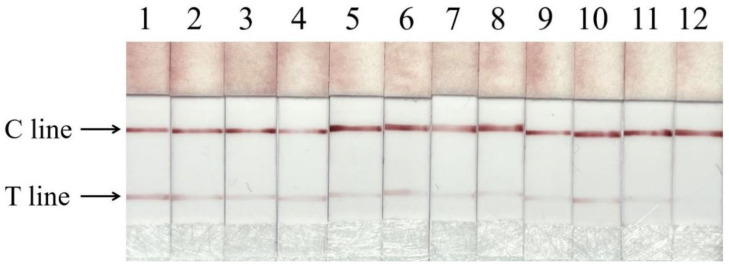
The Chicken-PCR-LFS system detects the adulteration of commercial samples. Numbers 1–11: positive sample test results; Numbers 12: negative control.

48 different types of beef skewers, beef rolls, beef meatballs, lamb skewers, and lamb rolls were purchased from the market to evaluate the performance of the PCR-LFS system. The results showed that 11 out of the 48 commercial samples were contaminated with chicken (Figure 7). Among them, there were 6 out of 30 commercial beef samples containing chicken ingredients, and 5 out of 18 commercial lamb samples containing chicken ingredients (Table 3). The above results show that our method can be used for the detection of commercially processed meat products and has good practical applications. Furthermore, we can achieve adulteration identification of different animal-origin foods by changing specific primers. Many PCR-based techniques have been reported for the detection of the adulteration of meat products [12,44]. Nevertheless, the long test times and the need for a PCR instrument make it unsuitable for non-laboratory environments. In recent years, isothermal amplification technology has been rapidly developed [45,46,47]. Compared with traditional PCR technology, isothermal amplification does not require expensive thermal cycling instruments, can amplify the target in a shorter period of time, and is suitable for on-site detection. However, isothermal amplification techniques have disadvantages, such as difficulty in primer design, vulnerability to aerosol contamination, and the high cost of detection requiring multiple enzymes, making them unsuitable for large-scale detection. This study combines highly sensitive PCR with a simple and intuitive flow measurement sensor, eliminating the need for electrophoresis and gel imaging equipment and providing an important tool for the identification of meat products.

## 4. Conclusions

This study proposed and developed an accurate, simple, and cost-effective detection strategy for chicken adulteration based on the PCR-LFS system. The inexpensive, environmentally friendly salt method can be used as an alternative to commercial kits to efficiently extract DNA at high concentrations and purity. High specificity of this system at an annealing temperature of 60 °C, without primer secondary structure and non-specific products. The system with good sensitivity to detect 0.1% (*w*/*w*) of chicken in beef, lamb and ostrich. In addition, the system has a high application value, as the PCR amplicons can be used directly in the test strip, and the results can be visualized by the naked eye within 5 min. In summary, the developed PCR-LFS strategy can be applied to the detection of meat adulteration in resource-limited areas.

## Figures and Tables

**Table 1:** Sample preparation to simulate chicken adulteration.

Chicken Adulteration Ratio	100%	10%	1%	0.1%	0
Beef mixed with chicken	300 mg chicken	270 mg beef + 30 mg chicken	297 mg beef + 3 mg chicken	299.7 mg beef + 0.3 mg chicken	300 mg beef
Lamb mixed with chicken	300 mg chicken	270 mg lamb + 30 mg chicken	297 mg lamb + 3 mg chicken	299.7 mg lamb + 0.3 mg chicken	300 mg lamb
Beef, lamb and ostrich meat mixed with chicken	300 mg chicken	90 mg beef + 90 mg lamb + 90 mg ostrich + 30 mg chicken	99 mg beef + 99 mg lamb + 99 mg ostrich + 3 mg chicken	99.9 mg beef + 99.9 mg lamb + 99.9 mg ostrich + 0.3 mg chicken	100 mg beef + 100 mg lamb + 100 mg ostrich

**Table 2:** Comparison of DNA concentration and purity six methods, DNA concentrations (ng/µL), A260/A280 and A260/A230 were measured with the NanoDrop 2000 nucleic acid analyzer.

Method	DNA Concentration ± SD ^1^ (ng µL^−1^)	Mean Ratio A260/A280	Mean Ratio A260/A230
CTAB method	70.0 ± 9.7 ^c^	1.5 ± 0.03	0.5 ± 0.10
Salt method	533 ± 84 ^a^	2.0 ± 0.09	2.8 ± 0.82
Urea method	314 ± 13 ^a,b^	1.9 ± 0.09	2.0 ± 0.19
SDS method	251 ± 58 ^b^	2.0 ± 0.01	2.4 ± 0.28
Guanidine isothiocyanate method	241 ± 75 ^b^	1.8 ± 0.15	1.8 ± 0.07
Kit method	289 ± 11 ^a,b^	2.0 ± 0.02	2.4 ± 0.34

^1^ SD—standard deviation, n = 3; ^a^, ^b^ and ^c^ are significantly different between the columns (*p* < 0.05).

**Table 3:** Identification results of commercially available samples.

Type	Name	Samples Number	Adulteration Number
Beef products	Beef skewers, beef rolls, beef meatballs	30	6
Lamb products	Lamb skewers, lamb rolls	18	5

## Data Availability

Not applicable.
